# Glioma consensus contouring recommendations from a MR-Linac International Consortium Research Group and evaluation of a CT-MRI and MRI-only workflow

**DOI:** 10.1007/s11060-020-03605-6

**Published:** 2020-08-29

**Authors:** Chia-Lin Tseng, James Stewart, Gillian Whitfield, Joost J. C. Verhoeff, Joseph Bovi, Hany Soliman, Caroline Chung, Sten Myrehaug, Mikki Campbell, Eshetu G. Atenafu, Chinthaka Heyn, Sunit Das, James Perry, Mark Ruschin, Arjun Sahgal

**Affiliations:** 1 Department of Radiation Oncology, Sunnybrook Health Sciences Centre, University of Toronto, 2075 Bayview Avenue, Toronto, ON M4N 3M5 Canada; 2 Manchester Academic Health Science Centre, University of Manchester, The Christie NHS Foundation Trust, Manchester, UK; 3 Department of Radiation Oncology, University Medical Center Utrecht, Utrecht, The Netherlands; 4 Department of Radiation Oncology, Froedtert Memorial Lutheran Hospital, Milwaukee, WI USA; 5 Department of Radiation Oncology, The University of Texas MD Anderson Cancer Center, Houston, TX USA; 6 Departments of Biostatistics, University Health Network, University of Toronto, Toronto, ON Canada; 7 Department of Medical Imaging, Sunnybrook Health Sciences Centre, University of Toronto, Toronto, ON Canada; 8 Division of Neurosurgery, St. Michael’s Hospital, University of Toronto, Toronto, ON Canada; 9 Department of Medicine, Division of Neurology, Sunnybrook Health Sciences Centre, University of Toronto, Toronto, ON Canada

**Keywords:** Consensus contouring recommendations, Glioma, Radiotherapy, Organs-at-risk, MR-linac

## Abstract

**Introduction:**

This study proposes contouring recommendations for radiation treatment planning target volumes and organs-at-risk (OARs) for both low grade and high grade gliomas.

**Methods:**

Ten cases consisting of 5 glioblastomas and 5 grade II or III gliomas, including their respective gross tumor volume (GTV), clinical target volume (CTV), and OARs were each contoured by 6 experienced neuro-radiation oncologists from 5 international institutions. Each case was first contoured using only MRI sequences (MRI-only), and then re-contoured with the addition of a fused planning CT (CT-MRI). The level of agreement among all contours was assessed using simultaneous truth and performance level estimation (STAPLE) with the kappa statistic and Dice similarity coefficient.

**Results:**

A high level of agreement was observed between the GTV and CTV contours in the MRI-only workflow with a mean kappa of 0.88 and 0.89, respectively, with no statistically significant differences compared to the CT-MRI workflow (p = 0.88 and p = 0.82 for GTV and CTV, respectively). Agreement in cochlea contours improved from a mean kappa of 0.39 to 0.41, to 0.69 to 0.71 with the addition of CT information (p < 0.0001 for both cochleae). Substantial to near perfect level of agreement was observed in all other contoured OARs with a mean kappa range of 0.60 to 0.90 in both MRI-only and CT-MRI workflows.

**Conclusions:**

Consensus contouring recommendations for low grade and high grade gliomas were established using the results from the consensus STAPLE contours, which will serve as a basis for further study and clinical trials by the MR-Linac Consortium.

**Electronic supplementary material:**

The online version of this article (10.1007/s11060-020-03605-6) contains supplementary material, which is available to authorized users.

## Introduction

Magnetic Resonance Imaging (MRI) has long been used for radiotherapy definition of brain tumors and organs-at-risk (OARs). In adult low and high grade gliomas, the current standard of care consists of maximal safe resection followed by radiotherapy with or without concurrent and/or adjuvant chemotherapy [1–6]. The standard practice has been to use post-gadolinium T1-weighted and T2-weighted or T2 fluid attenuation inversion recovery (FLAIR) sequences, which are fused to computed tomography (CT) for the delineation of target volumes and OARs. Although there are limitations of conventional MRI for target definition of gliomas, the precise roles of metabolic and physiological imaging (eg. proton MR spectroscopic imaging, chemical exchange saturation transfer imaging, perfusion imaging, water diffusion imaging) have yet to be established and are active areas of investigation [7].

Radiotherapy target volumes have differed among various trials group, particularly with respect to the clinical target volume (CTV) as an anatomic margin beyond that of the gross target volume (GTV). Inter-observer variability in target volume delineation of glioblastoma multiforme (GBM) has been evaluated and limited recommendations proposed with respect to target volumes and OARs in previous reports, in some cases in the absence of quantitative analyses [8–10]. With the emergence of MR-guided radiotherapy systems, there is increasing interest in an MRI-only workflow for radiotherapy simulation and planning [7]. The improvements in immobilization and image guidance techniques underscore the critical importance of accurate and consistent target volume and OAR delineation. Within the context of the MR-Linear Accelerator (MR-Linac) International Consortium Research Group [11], the aim of the present study is to develop contouring recommendations in low and high grade gliomas for targets and OARs based on consensus contours for both CT-MRI and MRI-only workflows, and to identify any differences in agreement between the two workflows. This represents an international effort and will serve as the basis of future collaborative clinical trials to ensure uniformity in glioma contouring.

## Methods and Materials

Ten cases of glioma consisting of 5 GBM and 5 WHO grade II or III gliomas were selected from a prospective institutional database of patients for this study. The study was approved by the institutional ethics review board. No informed consent was required as per retrospective and anonymized nature of the image datasets. The cases were selected with the intent to represent varying tumor size, locations within the brain, and proximity to OARs as well as white matter pathways. Six international radiation oncology experts from 5 institutions, who treat gliomas in clinical practice and/or have reported clinical series, participated in the present study. For each case, a post-operative volumetric post-gadolinium T1-weighted MRI and fused T2/FLAIR MRI were provided to each participant. For the MRI-only workflow, each participant was given access only to the MRIs to complete contours of the GTV, CTV, and OARs including lenses, globes, optic nerves, optic chiasm, brainstem, and cochlea. For the CT-MRI workflow, the participants were then asked to re-contour all structures on each of the ten cases with the additional information provided by a fused non-contrast enhanced 1-mm slice thickness planning CT. CTV expansion was 1.5 cm from enhancing disease on post-gadolinium T1-weighted MRI for GBM cases and 1.0 cm from T2/FLAIR MRI hyperintense disease for WHO grade II and III glioma cases. A single phase/volume approach was used in defining the GTV and CTV in all cases [9]. The OAR contour definitions provided to each participant were consistent with prior published guidelines [9, 12].

All cases were contoured in the Monaco treatment planning system (TPS) version v5.19.03 (Elekta AB, Stockholm, Sweden). The first 4 cases were completed at each of the participant’s respective institutions via a cloud-based TPS installation, and the latter 6 cases were completed on-site at the coordinating institution after a consensus meeting/workshop. All completed contours were transferred from the TPS to ADMIRE segmentation software version v2.0.0.1 (Elekta AB, Stockholm, Sweden) and to analysis software MATLAB, version 2016b (MathWorks, Natick, MA). Contour agreement was measured by combinatorial pair-wise comparisons of the Dice similarity coefficient (DSC) [13].

Consensus contours were generated for each case using an expectation–maximization algorithm for simultaneous truth and performance level estimation (STAPLE). [14, 15] Kappa (к) statistic was calculated to quantify agreement between participant contours, where к < 0 signifies less agreement than would be expected by chance, 0 to 0.20, none to slight agreement; 0.21 to 0.40, fair agreement; 0.41 to 0.60, moderate agreement; 0.61 to 0.80, substantial agreement; and 0.81 to 1.00, almost perfect agreement [16]. Contour agreement before (cases 1 to 4) and after (cases 5 to 10) the on-site consensus meeting/workshop were compared. A mixed model analysis was used for DSC and kappa statistic to account for collinearity, as the contouring data was obtained from the same images. All P values were 2-sided, and P < 0.05 was considered to indicate a significantly different result. Statistical analyses were performed using version 9.4 of the SAS system for Windows (2002–2012; SAS Institute, Cary, NC). All participants then reviewed the STAPLE contours to identify any discrepancies and following two review meetings, consensus contours were finalized and recommendations formed for GTV, CTV, and OAR delineation.

## Results

**Fig. 1:**
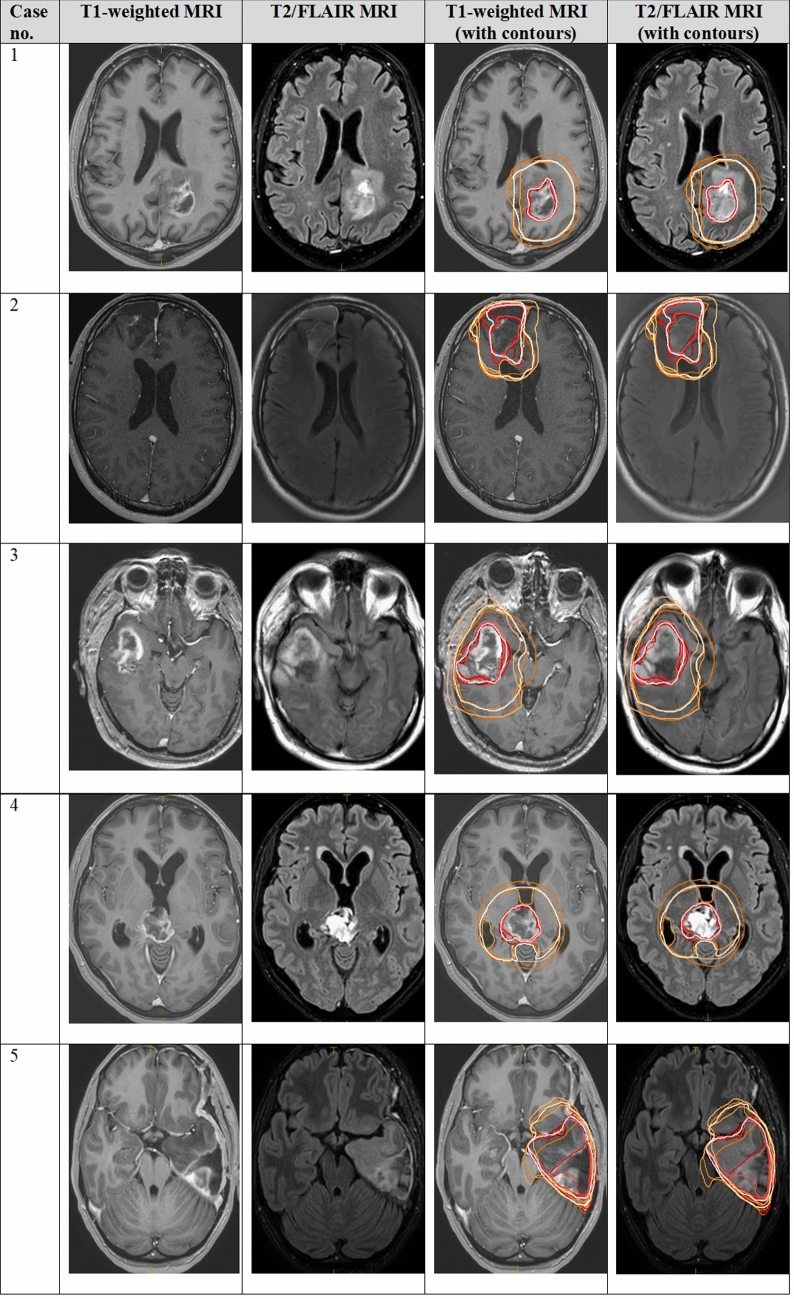
Individual physician and consensus target volume contours on selected axial slice for the GTV and CTV in low and high grade gliomas, cases 1–5. Consensus contours are shown in thick white and individual contours in shades of other colors. *MRI* magnetic resonance imaging, *GTV* gross tumor volume, *CTV* clinical target volume

**Fig. 2:**
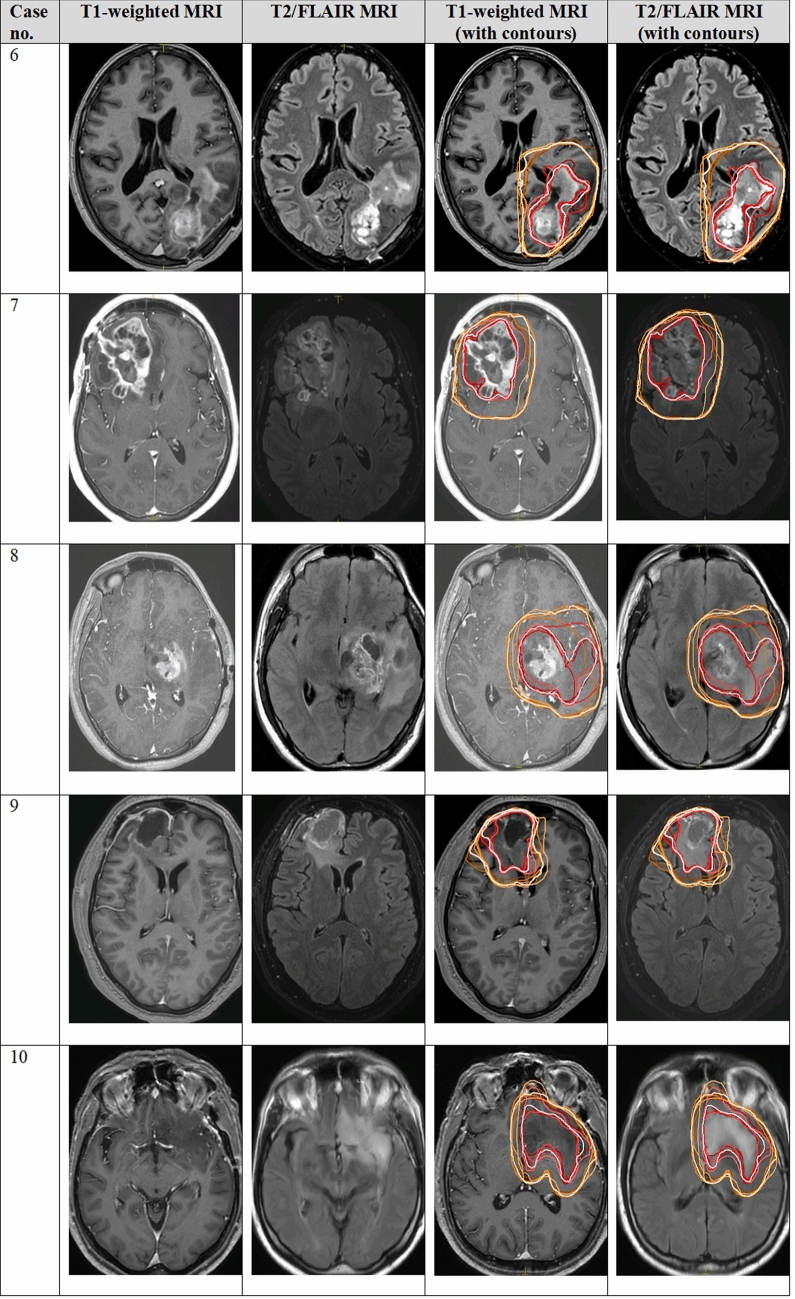
Individual physician and consensus target volume contours on selected axial slice for the GTV and CTV in low and high grade gliomas, cases 6–10. Consensus contours are shown in thick white and individual contours in shades of other colors. *MRI* magnetic resonance imaging, *GTV* gross tumor volume, *CTV* clinical target volume

Twenty sets (10 for MRI-only workflow, 10 for CT-MRI workflow) of GTV, CTV, and OAR contours corresponding to the 10 glioma cases were completed by each of the 6 participating physicians. In total 236 target contours and 1154 OAR contours were analyzed. No contours were excluded for analysis; however, missing contours were taken into account during analysis. The case descriptions are summarized in Table 1. Figures 1 and 2 illustrate the expert contours and STAPLE consensus contours of the GTV and CTV on a representative axial slice of contrast-enhanced T1-weighted and T2/FLAIR MRI for all 10 cases. Of the study cases, two involved the thalamus, one involved the brainstem, five crossed midline via a white matter tract pathway, and two involved the ventricles and/or ependyma.

**Table 1:** Case description

Case number	Description
1	GBM, left parietal
2	Grade 2 oligodendroglioma, IDH mutated, 1p19q co-deleted, right frontal
3	GBM, right parieto-temporal
4	GBM, thalamic/pineal
5	Grade 2 astrocytoma, IDH mutated, no 1p19q co-deletion, ATRX loss, left temporo-parietal
6	GBM, left occipital with ependymal extension to the left lateral ventricle
7	GBM, right fronto-temporal
8	Grade 3 oligodendroglioma, 1p/19q co-deleted, no conventional IDH mutation, left fronto-temporal/brainstem
9	Grade 3 astrocytoma, (progression from prior grade 2 astrocytoma, IDH mutated), right frontal
10	Grade 2 oligodendroglioma, IDH mutated, 1p/19q co-deleted, left frontal/thalamic

### Analysis of target volumes (GTV and CTV)

The results of the STAPLE and DSC analysis for the GTVs and CTVs for the 10 cases, each with two workflows (MRI-only, CT-MRI), are summarized in Table 2. Overall, a very high level of agreement was observed among the participating physicians for both GTV and CTV. For the MRI-only workflow, the mean kappa was 0.88 and 0.89 for the GTV and CTV, respectively. For the CT-MRI workflow, the mean kappa was 0.88 and 0.89 for the GTV and CTV, respectively. This corresponds to very good agreement, with no statistically significant difference in agreement between the two workflows (p = 0.88 and p = 0.82, for GTV and CTV, respectively). Similarly, the DSC analysis demonstrated high concordance of the contoured volumes among the participants with a mean DSC range of 0.86 to 0.88 for GTV and CTV. No kappa below 0.83 was observed in any of the 10 cases.

**Table 2:** STAPLE and dice similarity coefficient (DSC) analysis for target volumes (GTV and CTV) for each of the 10 cases among participating physicians

Case no	Mean SENS		Mean SPEC		Mean Kappa (к)			Mean DSC		
	MRI-Only	CT-MRI	MRI-Only	CT-MRI	MRI-Only	CT-MRI	P-value	MRI-Only	CT-MRI	P-value
1 GTV	0.95	0.95	0.99	0.99	0.94	0.94	0.36	0.93	0.93	< 0.01
CTV	0.96	0.97	0.96	0.96	0.88	0.89	0.48	0.88	0.89	0.48
2 GTV	0.85	0.85	0.98	0.99	0.83	0.84	0.24	0.80	0.81	< 0.01
CTV	0.92	0.91	0.98	0.98	0.88	0.88	0.98	0.86	0.86	0.49
3 GTV	0.95	0.95	0.99	0.99	0.93	0.93	0.36	0.93	0.93	0.04
CTV	0.97	0.98	0.96	0.96	0.88	0.89	0.73	0.88	0.89	0.83
4 GTV	0.92	0.92	0.99	0.99	0.90	0.90	0.36	0.90	0.90	0.06
CTV	0.92	0.92	0.95	0.96	0.83	0.84	0.41	0.81	0.83	1.00
5 GTV	0.82	0.82	0.99	0.99	0.83	0.83	0.36	0.77	0.76	0.16
CTV	0.88	0.88	0.98	0.98	0.87	0.87	0.82	0.84	0.84	0.40
6 GTV	0.91	0.91	0.98	0.98	0.87	0.87	0.08	0.83	0.83	0.16
CTV	0.96	0.95	0.98	0.98	0.91	0.91	0.53	0.90	0.90	0.78
7 GTV	0.95	0.95	0.99	0.99	0.93	0.93	0.79	0.92	0.92	0.62
CTV	0.94	0.94	0.99	0.99	0.92	0.92	0.70	0.92	0.92	0.55
8 GTV	0.89	0.89	0.98	0.98	0.87	0.87	0.17	0.85	0.85	0.19
CTV	0.93	0.93	0.98	0.98	0.90	0.90	0.66	0.90	0.90	0.77
9 GTV	0.90	0.90	0.98	0.98	0.87	0.87	0.14	0.85	0.85	< .0001
CTV	0.93	0.93	0.98	0.98	0.89	0.90	0.23	0.89	0.89	0.01
10 GTV	0.88	0.89	0.99	0.99	0.88	0.88	0.22	0.85	0.85	0.04
CTV	0.91	0.92	0.99	0.99	0.89	0.90	0.12	0.88	0.88	< 0.01

*SENS* STAPLE sensitivity, *SPEC* STAPLE specificity, *DSC* dice coefficient, *MR* magnetic resonance imaging, *CT* computed tomography

### Analysis of OARs

The results of the STAPLE and DSC analysis for the OARs for the 10 cases, each with two workflows (MRI-only, CT-MRI), are summarized in Table S1. A moderate to high level of agreement was observed among the participants for all OARs with the exception of the cochlea in the MRI-only workflow. The mean kappa for all OARs except the optic chiasm and cochlea ranged from 0.74 to 0.90 and 0.73 to 0.90, for the MRI-only and CT-MRI workflow, respectively. This corresponds to at least substantial agreement, with no statistically significant difference in agreement between the two workflows. The largest variability was seen in the contouring of cochlea in the MRI-only workflow with a mean kappa range of 0.39 to 0.41. This improved significantly with the addition of a fused planning CT to 0.69 to 0.71 (p < 0.0001). The DSC analysis demonstrated similar results to the STAPLE analysis as shown in Table S1.

### Consensus meeting and contouring recommendations

Contour agreement of all structures before (cases 1 to 4) and after (cases 5 to 10) the on-site consensus meeting/workshop was compared to assess the effect of diagnostic on-site radiology-led didactic sessions of anatomy relevant to glioma contouring and consensus discussions. The results of the comparisons are summarized in Table S2. Of note, a statistically significant improvement in mean kappa statistic was observed in the contouring of cochlea in the CT-MRI workflow (p = 0.02 to 0.045), and of globes in both the MRI-only and CT-MRI workflows (p = 0.005 to 0.04).

The STAPLE contours were then reviewed during two follow-up consensus meetings of participating physicians. Discussions were held to address areas of discrepancy in contouring within the clinical context of each case. Once agreement had been reached, consensus contours were generated and 6 recommendations were developed to guide CTV delineation as summarized in Table 3.

**Table 3:** Recommendations for CTV contouring of low and high grade gliomas

Recommendations	
1	In the absence of contiguous white matter tracts, the CTV should be limited, without additional margin, by the following anatomical barriers: falx, tentorium cerebelli, and inner table of the skull
2	The brainstem is an anatomical barrier when the enhancing or T2/FLAIR hyperintense tumor is not situated along a contiguous white matter pathway; however, for tumors located in adjacent structures along white matter tracts (i.e. thalamus, internal capsule), the CTV should extend into the brainstem (whether the ipsilateral half or entire brainstem need to be taken in the CTV expansion is not well defined)
3	The CTV should be limited by, without additional margin, the optic nerves and chiasm; however, the optic tracts (+/− chiasm/optic nerves) should not be excluded from the CTV when the GTV is in contiguity anatomically with the optic structures
4	The CTV does not need to be excluded from the ventricles, and should be included in event of ependymal or leptomeningeal involvement
5	The CTV should cross into the contralateral hemisphere if the enhancing or T2/FLAIR hyperintense tumor encroaches on the following white matter tracts: corpus callosum (genu and splenium), anterior commissure (inferior to the frontal horns and superior the third ventricle), and posterior commissure (dorsal to the cerebral aqueduct)
6	The interthalamic adhesion is present in most human brains, and consideration should be given to extend the CTV into the contralateral thalamus if the enhancing or T2/FLAIR hyperintense tumor encroaches on the medial thalamus

## Discussion

The present study has generated detailed recommendations for CTV contouring in low and high grade gliomas including GBM to guide clinical practice, based on consensus contours among international experts within the MR-Linac International Consortium Research Group (Table 3). Moreover, we investigated the inter-observer variability and the effect of an MRI-only workflow vs. the traditional CT-MRI workflow. Overall, the results demonstrated excellent nonrandom agreement among the contouring physicians for GTV and CTV, with a mean kappa range of 0.88 to 0.89 for both the MRI-only and CT-MRI workflows (Table 2).

The results from our study compare favorably to other glioma delineation studies. For example, the Korean Radiation Oncology Group observed considerable variability amongst 15 radiation oncologists’ contours of GTVs and CTVs (mean kappa 0.58 and 0.65, respectively) in 9 cases of newly diagnosed GBM patients [10], and the recently reported NRG consensus contouring study by 10 radiation oncologists of 4 GBM cases using a two-dose-level approach reported a kappa statistics range from 0.59 to 0.81 for high and low dose GTVs, and 0.72 to 0.85 for high and low dose CTVs [8]. In our study, the largest variability was observed in case 4, which illustrated a pineal/thalamic GBM with inferior extension to the superior midbrain and posterior abutment of the tentorium cerebelli. The differences in contouring arose from the lack of inclusion of ventricular spaces by one participant, and failure to trim the CTV from the posterior fossa in another. These omissions were recognized and discussed in the consensus meetings, and included in the recommendations presented in Table 3.

The consensus STAPLE contour for each CTV underscores several important observations. First, the CTV expansion is limited, in the absence of contiguous white matter tracts, to anatomic barriers of spread including the falx, tentorium cerebelli and the inner table of the skull. The brainstem is an anatomical barrier only when the enhancing or T2/FLAIR hyperintense tumor is not situated along a contiguous white matter pathway (e.g. thalamus, internal capsule). By the same principle, the CTV should be limited by, without additional margin, the optic nerves and chiasm but the optic tracts should not be excluded from the CTV (e.g. cases 3, 5, 7, 8, 10). Commissural tracts connect opposing cerebral hemispheres and, thus, the CTV should cross into the contralateral hemisphere if the enhancing or T2/FLAIR hyperintense tumor encroaches on the corpus callosum (genu and splenium), anterior commissure, and posterior commissure (e.g. cases 1, 2, 7, 9, 10). Finally, the interthalamic adhesion consist of a small bridge of tissue between the thalami, which is not always present in healthy human volunteers [17]. Animal studies indicate that the interthalamic adhesion may be important in communication across cerebral hemispheres and gliomatosis cerebri [18, 19]; however, its functional significance in humans is unknown [20]. The investigators agree that as the interthalamic adhesion is present in most human brains (~ 80%), consideration should be given to extend the CTV into the contralateral thalamus if the enhancing or T2/FLAIR hyperintense tumor encroaches on the medial thalamus (e.g. case 8).

The results from the current study demonstrate at least substantial agreement (mean kappa range from 0.74 to 0.90 and 0.73 to 0.90, for the MRI-only and CT-MRI workflow, respectively) for all OARs with the exception of the optic chiasm and cochlea. The observed differences in optic chiasm contouring may be due to the challenges in identifying the anatomical borders of the structure in relation to its transition into optic nerves anteriorly and optic tracts posteriorly. Similarly, Sanstrom et al. reported large variability in the contouring of the optic chiasm in a study evaluating OAR contouring practices in international radiosurgery institutions [21]. In cases (e.g. cases 3, 5, 7, 8, 10) where the GTV lies adjacent to the optic chiasm and/or tracts, this may translate into a wide variation in maximum doses to the optic apparatus as it falls within a region of high dose gradient falloff. For consistency, a 5 mm extension along the anterior and posterior limbs of the optic chiasm is recommended, as defined in the ESTRO-ACROP guideline [9], while avoiding gaps to ensuring continuity of the entire optic pathway. The cochleae are best visualized with a heavily T2-weighted thin-slice MRI through the internal auditory canal (IAC) or a high-resolution CT of the temporal bone to identify the fluid-filled spaces of the membranous labyrinth. Therefore, it was unsurprising that the MRI-only workflow showed a poor level of agreement with a mean kappa range of 0.39 to 0.41, which improved significantly with the addition of a fused planning CT to 0.69 to 0.71 (p < 0.0001). This underscores the potential benefit of using a synthetic CT for contouring purposes in an MRI-only workflow [22]. For all other OARs no statistically significant differences were demonstrated in contour variability between the MRI-only workflow and CT-MRI workflow, confirming the feasibility of an MRI-only workflow. Moreover, the educational benefit of an on-site consensus meeting/workshop was illustrated through a statistically significant improvement in mean kappa statistic in the contouring of cochlea in the CT-MRI workflow (p = 0.02 to 0.045).

For GBM radiotherapy, currently two major approaches exist, supported by the European Organization for Research and Treatment of Cancer (EORTC) and the Radiotherapy and Oncology Group (RTOG/NRG), respectively. The EORTC recommends a single-phase/volume approach. In contrast, the RTOG/NRG recommends a two-phase/volume approach wherein the second phase/volume consists of a “cone-down” or boost to a smaller target volume. At this time, no consensus exists within the radiation oncology community with respect to the optimal approach. It is recognized that glioma extends along white matter tract pathways within the brain parenchyma, and older autopsy and histopathologic correlative studies suggest that tumor cells may be found even beyond regions of abnormal signal depicted on post-contrast T1-weighted and T2-weighted MRIs. [23, 24] However, published retrospective studies have shown that the majority of failures tend to be within the enhancing central tissue, and no differences in pattern of failure have been observed between the EORTC and RTOG/NRG target volume definitions [25, 26]. The reported ESTRO-ACROP guideline for target delineation of GBM recommends a CTV defined by a margin of 2 cm beyond that of GTV, although it acknowledges that a range of margins have been allowed on various EORTC clinical trials [9]. On the other hand, a number of studies have suggested that a reduction in CTV margin may not alter the pattern of failure in GBM, as most failures tend to be in-field within the high dose volume. Such margin reduction and, thus, treated volume exposed to high dose radiation may translate into potential reduction in toxicities [25–29]. Therefore, for consistency of delineation, the current study utilizes a 1.5 cm and 1.0 cm margin for CTV in GBM and grade II or III gliomas, respectively, while respecting anatomic barriers. This definition is consistent with those proposed by reported multi-center phase III GBM and high-risk low grade glioma studies [6, 30]. Table S3 summarizes a comparison of the key findings and recommendations proposed in published reports of GBM consensus contouring, including the present study. We observed that Korean Radiation Oncology Group did not propose specific recommendations with regards to CTV modifications in relation to the surrounding anatomy although adjustments were made with varying frequency depending on the adjacent structure: the falx (80%), the tentorium (71%), and the ventricular system (34%) [10]. Furthermore, the ESTRO-ACROP GBM guideline, although providing a detailed expert consensus of target delineation, lacked quantitative analyses of inter-observer contour variability [9]. Finally, Kruser et al. recently reported an NRG brain tumor specialist consensus report on GBM contouring, which highlighted several similar findings with respect to limiting the CTV to anatomical boundaries such as the falx, the tentorium cerebelli, and extension of the CTV across the commissural pathways and into the brainstem [8]. However, the NRG study was limited by only a few study cases (4 GBM cases in total) and, therefore, tumor locations. Moreover, comparison of CTV volumes with the present study’s recommendations is difficult given the two-phase approach adopted in the NRG study. Ultimately, the appropriate target volume definition is a balance between a thorough understanding of the anatomic pathways of spread, the anatomic barriers (falx, tentorium cerebelli, bone), and minimization of toxicities to adjacent OAR.

A key strength in the present study is the large number of study cases intentionally selected to represent variation in tumor location and proximity to surrounding anatomy. Our study evaluated both target volumes (GTV, CTV) and OAR commonly delineated in glioma radiation planning, and a high level of agreement was observed in nearly all cases. Furthermore, this study is the only report to date investigating both CT-MRI and MRI-only workflows with an analysis to assess any differences in agreement between the two workflows. With the recent development of MR-guided radiotherapy systems, specifically integrated MRI-linear accelerator systems, the precision of radiation treatment could be improved through daily MRI and planning streamlined with the implementation of an MRI-only workflow [7]. The potential benefits of an MR-Linac are multifold. First, an MRI-only workflow could be time- and cost-saving while minimizing uncertainty associated with CT-MRI registration. Further, MRI-based treatment delivery workflow allows direct visualization of normal tissues and tumor-related changes that cannot be adequately appreciated on CT to prompt anatomical based adaptations throughout the course of therapy. Most importantly, an MR-Linac introduces the potential for functional image acquisition such as diffusion, chemical exchange saturation transfer (CEST), perfusion, and other quantitative MRI (qMRI) biomarkers to facilitate early outcome prediction and thereby individualized patient selection for treatment modifications [31–36]. It cannot be underscored enough that consistent contouring approaches for all target and OAR structures in glioma is critical in an adaptive strategy based on MR-guided radiotherapy, facilitates radiomics in glioma research [37], and underpins interpretation of dosimetric and clinical outcomes in future collaborative studies.

It is recognized that several limitations exist within the current study. First, although the cases were selected to represent a wide range of clinical scenarios and tumor locations, the recommendations may not be applicable to all situations, and unique circumstances will require the clinical expertise and judgment of the treating physicians. Second, it is not the intent nor within the scope of the present study to address the CTV margin expansion as a range of margins has been applied to the GTV in clinical trials for low and high grade gliomas, although the margins used in this study are consistent with pattern-of-failure data and recent clinical trial protocols.

## Conclusions

In conclusion, our study demonstrates that the addition of CT to an MRI-only workflow does not provide additional anatomical information in gliomas to significantly reduce inter-observer contouring variability with the exception of the cochlea. Dedicated MRI sequences may be required for consistent delineation of the cochlea when inclusion of the OAR is indicated. Consensus contouring recommendations for CTV in low grade and high grade gliomas were established, supported quantitatively by a high level of agreement, which represents an important contribution in consistent delineation of targets. This will serve as a basis for further investigation, in particular collaborative studies in the context of emerging MRI-only workflow strategies within the international MR-Linac consortium.

## Electronic supplementary material

Below is the link to the electronic supplementary material.

Supplementary file1 (PDF 1350 kb)
