# Supplementary material for: Glioma consensus contouring recommendations from a MR-Linac International Consortium Research Group and evaluation of a CT-MRI and MRI-only workflow
Source: J Neurooncol. 2020 Aug 29;149(2):305–14. doi: 10.1007/s11060-020-03605-6 (PMC7541359; doi:10.1007/s11060-020-03605-6)
Supplement: Supplementary file 1 — Supplementary file1 (PDF 1350 kb) [file 11060_2020_3605_MOESM1_ESM.pdf]

Table S1. Summary STAPLE and Dice similarity coefficient (DSC) analysis for organs-at-risk (OARs) for all 10 cases among participating physicians

Organs-at-risk (OAR)	Mean SENS		Mean SPEC		Mean Kappa (κ)			Mean DSC		
	MRI-Only	CT-MRI	MRI-Only	CT-MRI	MRI-Only	CT-MRI	P-value	MRI-Only	CT-MRI	P-value
Brainstem	0.91	0.92	0.99	0.99	0.90	0.90	0.99	0.88	0.88	0.98
Optic Chiasm	0.58	0.58	1.00	1.00	0.60	0.60	0.97	0.49	0.49	0.92
Globe (L)	0.93	0.93	0.99	0.99	0.89	0.89	0.87	0.89	0.89	0.70
Globe (R)	0.93	0.93	0.99	0.99	0.90	0.89	0.93	0.90	0.90	0.89
Lens (L)	0.76	0.76	1.00	1.00	0.77	0.77	0.86	0.72	0.71	0.45
Lens (R)	0.73	0.73	1.00	1.00	0.74	0.74	0.92	0.69	0.69	0.70
Cochlea (L)	0.43	0.68	0.99	1.00	0.39	0.71	< 0.0001	0.17	0.62	< 0.0001
Cochlea (R)	0.42	0.67	0.99	1.00	0.41	0.69	< 0.0001	0.20	0.59	< 0.0001
Optic Nerve (L)	0.68	0.67	1.00	1.00	0.75	0.74	0.71	0.65	0.64	0.34
Optic Nerve (R)	0.67	0.67	1.00	1.00	0.74	0.73	0.98	0.63	0.63	0.91

Abbreviations: SENS = STAPLE sensitivity; SPEC = STAPLE specificity; DSC = Dice coefficient; MRI = magnetic resonance imaging; CT = computed tomography; L = left; R = right

Table S2. Comparison of contour agreement pre- (cases 1-4) and post- (cases 5-10) on-site consensus meeting/workshop

Organs-at-risk (OAR)	Kappa (κ) MRI-Only Workflow			Kappa (κ) CT-MRI Workflow		
	Pre-Meeting Mean	Post-Meeting Mean	P-value	Pre-Meeting Mean	Post-Meeting Mean	P-value
GTV	0.90	0.87	0.28	0.90	0.87	0.22
CTV	0.86	0.90	0.07	0.87	0.90	0.16
Brainstem	0.91	0.89	0.28	0.91	0.90	0.29
Optic Chiasm	0.58	0.61	0.67	0.58	0.61	0.63
Globe (L)	0.87	0.90	0.01	0.87	0.90	0.005
Globe (R)	0.88	0.91	0.04	0.88	0.91	0.03
Lens (L)	0.78	0.77	0.85	0.76	0.77	0.89
Lens (R)	0.72	0.75	0.44	0.72	0.75	0.35
Cochlea (L)	0.34	0.42	0.40	0.64	0.74	0.045
Cochlea (R)	0.34	0.46	0.06	0.60	0.73	0.02
Optic Nerve (L)	0.74	0.75	0.82	0.73	0.75	0.47
Optic Nerve (R)	0.72	0.74	0.47	0.72	0.74	0.49

Table S3. Comparison of clinical target volume (CTV) findings/recommendations among published reports for glioblastoma multiforme (GBM)

Study	Margin from Gross Tumor Volume (GTV)	Anatomical Barrier(s) of Spread	Brainstem Extension	Optic Pathway Extension	Commissural Extension
Present study	1.5 cm beyond surgical cavity and residual enhancing disease for GBM	CTV should be limited, without additional margin, by the falx, tentorium cerebelli, and inner table of the skull; CTV does not need to be excluded from the ventricles, and should be included in event of ependymal or leptomeningeal involvement	No margin when enhancing tumor not situated along a contiguous white matter pathway; for tumors along white matter tracts (i.e. thalamus, internal capsule), CTV should extend into the brainstem (ipsilateral half or entire brainstem not well defined)	No margin at the optic nerves and chiasm; the optic tracts should not be excluded from the CTV when the GTV is in contiguity anatomically	CTV should cross into the contralateral hemisphere if the enhancing tumor encroaches on: corpus callosum (genu and splenium), anterior and posterior commissure; consider extension into the contralateral thalamus if enhancing tumor encroaches on medial thalamus
Wee et al.[10]	1.5 cm (median) corresponding to minimal margins needed to cover at least 95% of CTV_{STAPLE}	Not specified; constrain to potential barriers such as falx, tentorium, ventricular space	N/A	N/A	N/A
Niyazi et al. (ESTRO-ACROP)[9]	2.0 cm recommended; no definite recommendation on inclusion of regions of abnormal T2/FLAIR	Reduced margins at anatomical barriers at the skull (0 mm), ventricles (5 mm), falx (5 mm), tentorium cerebelli (5 mm), provided tumor is distant from white matter tracts extending to these regions	No margin (0 mm), provided the tumor is distant from white matter tracts extending to it	No margin (0 mm), provided the tumor is distant from white matter tracts extending to it	N/A
Kruser et al.	CTV_4600 2 cm beyond both	Trim from infratentorial	CTV_4600 includes 2 cm	CTV_4600 includes 2 cm	Avoid over-aggressive

(NRG)[8]	cavity/T1c-enhancement and T2/FLAIR hyperintensity; CTV_6000 2 cm beyond cavity/T1c-enhancement	brain for supratentorial tumors; trim CTV expansion directly to the falx (no margin beyond)	from T2-FLAIR if anatomic contiguity; CTV_6000 includes 2 cm from T1-contrast if anatomic contiguity (cover to 54 Gy); Gross disease included without margin in CTV_6000	from T2-FLAIR if anatomic contiguity; CTV_6000 includes 2 cm from T1-contrast if anatomic contiguity (cover to 54 Gy); Gross disease included (allowing chiasm PRV up to 60 Gy)	trimming at the level of the corpus callosum
